# Mitochondrial pleomorphy in plant cells is driven by contiguous ER dynamics

**DOI:** 10.3389/fpls.2015.00783

**Published:** 2015-09-24

**Authors:** Erica-Ashley Jaipargas, Kiah A. Barton, Neeta Mathur, Jaideep Mathur

**Affiliations:** Laboratory of Plant Development and Interactions, Department of Molecular and Cellular Biology, University of GuelphON, Canada

**Keywords:** mitochondria, pleomorphy, sugar, light, hypoxia, fission, endoplasmic reticulum

## Abstract

Mitochondria are pleomorphic, double membrane-bound organelles involved in cellular energetics in all eukaryotes. Mitochondria in animal and yeast cells are typically tubular-reticulate structures and several micro-meters long but in green plants they are predominantly observed as 0.2–1.5 μm punctae. While fission and fusion, through the coordinated activity of several conserved proteins, shapes mitochondria, the endoplasmic reticulum (ER) has recently been identified as an additional player in this process in yeast and mammalian cells. The mitochondria-ER relationship in plant cells remains largely uncharacterized. Here, through live-imaging of the entire range of mitochondria pleomorphy we uncover the underlying basis for the predominantly punctate mitochondrial form in plants. We demonstrate that mitochondrial morphology changes in response to light and cytosolic sugar levels in an ER mediated manner. Whereas, large ER polygons and low dynamics under dark conditions favor mitochondrial fusion and elongation, small ER polygons result in increased fission and predominantly small mitochondria. Hypoxia also reduces ER dynamics and increases mitochondrial fusion to produce giant mitochondria. By observing elongated mitochondria in normal plants and fission-impaired Arabidopsis *nmt1-2* and *drp3a* mutants we also establish that thin extensions called matrixules and a beads-on-a-string mitochondrial phenotype are direct consequences of mitochondria-ER interactions.

## Introduction

The term mitochondrion (Greek-mitos = thread; chondrion = grain) introduced by Benda (1899) recognized the peculiar thread-grain nature of these organelles and their tendency to morph quickly from one form to another. Lewis and Lewis (1915) provided meticulous descriptions of the dynamic behavior of mitochondria involving continuous contortions and undulatory movements that lead to changes in their morphology and position within the living cell. Today mitochondria are recognized as double membrane-bound organelles of an endo-symbiogenic origin that constitute major sites of oxygen consumption and ATP production in all eukaryotes. It has been unequivocally established that mitochondria undergo fission and fusion in order to maintain the optimal gradients required for driving energy production (Cavers, 1914; Bereiter-Hahn and Vöth, 1994; Nunnari et al., 1997; Bleazard et al., 1999; Welchen et al., 2014). Alterations in mitochondrial form thereby reflect the continuous flux in cellular energy that occurs due to fluctuations in temperature, oxygen, and carbon availability, and various metabolic processes (Hackenbrock, 1968; Hackenbrock et al., 1971; Novikoff and Holtzman, 1976; Bereiter-Hahn and Vöth, 1994).

The inner mitochondrial membrane (IMM) is the seat of the electron transport chain (ETC) and oxidative phosphorylation reactions and responds to fluctuating ATP/ADP levels. The relative arrangements of mitochondrial cristae and matrix change under different conditions as well. There are two general states that these arrangements fall under: orthodox or condensed. The orthodox state is characteristic of ATP-enriched, motile mitochondria and is indicated by an expanded matrix. By contrast the condensed state is exhibited by relatively immobile ADP-enriched mitochondria with electron dense matrix and dilated inter-cristal regions (Hackenbrock et al., 1971; Bereiter-Hahn and Vöth, 1994; Logan and Leaver, 2000; Logan, 2006a). Whereas, different eukaryotic cell types and even similar cells within an organism exhibit mitochondria of different shapes and sizes (Youle and van der Bliek, 2012) the predominant mitochondrial form observed in a majority of cells in an organism is usually presented as being typical for that particular organism. For example, the typical mitochondrion is described as long and filamentous in animal fibroblasts, whereas hepatocytes have a predominance of spherical or ovoid mitochondria (Youle and van der Bliek, 2012). While in yeast cells a tubular-reticulate form comprising of 1–10 mitochondria is considered typical (Hoffman and Avers, 1973; Bereiter-Hahn and Vöth, 1994; Nunnari et al., 1997; Sesaki and Jensen, 1999), in green plants they predominantly appear as discrete, spherical to ovoid, punctate organelles with diameters ranging from 0.2 to 1.5 μm (Matzke and Matzke, 1986; Köhler et al., 1997; Logan and Leaver, 2000; Arimura et al., 2004; Logan, 2006a). Interestingly, the first depiction of mitochondria in plant cells had shown elongated forms (Meves, 1904) which was reinforced by several subsequent light microscopy based studies (Gunning and Steer, 1986; Lichtscheidl and Url, 1990). Other investigations have revealed that mitochondria become abnormally enlarged and vermiform under certain conditions (Stickens and Verbelen, 1996; Logan and Leaver, 2000; Van Gestel and Verbelen, 2002; Logan, 2006b; Seguí-Simarro et al., 2008). These observations clearly indicate that as in other organisms, mitochondria in plants are also capable of becoming elongated and vermiform and some specific but as yet undefined conditions must cause and maintain their fission into small units. The first objective for our investigations was therefore to identify a common condition that might explain the punctate morphology of mitochondria in plant cells.

In animal cells, normally elongated mitochondria become small or fragmented under hyperglycemic conditions (Yu et al., 2008; Jhun et al., 2013). This led to the hypothesis that a similar sugar-induced phenomenon occurs in plant cells. In addition, a contextual requirement was to understand the cell-biological mechanism through which an elongated mitochondrion in a plant cell may become small. The widely accepted present-day view on the mechanism underlying mitochondria shape and motility involves several highly conserved genes (Logan et al., 2004; Scott et al., 2006; Arimura et al., 2008). Whereas, homologs for all the proteins implicated in mitochondrial fission in yeast and animal cells have not been identified so far in plants, at least two major factors have been well-characterized. These are Fission1, a tail-anchored membrane protein (hFis1 in mammals: Yoon et al., 2003; Stojanovski et al., 2004; Fis1p in yeast: Mozdy et al., 2000; Tieu and Nunnari, 2000; Fis1/BIGYIN in plants: Scott et al., 2006) and a mechano-chemical GTPase Dnm1 (Dnm1 in yeast: Bleazard et al., 1999; Dlp1 in mammals: Pitts et al., 1999; DRP3/ADL2 in plants: Arimura et al., 2004; Logan et al., 2004). The possibility of NETWORK MITOCHONDRIA/ELONGATED MITOCHONDRIA1 (NMT1/ELM1) being a functional plant homolog of the yeast Mdv1/Caf4 proteins has also been suggested (Logan et al., 2003; Arimura et al., 2008; Logan, 2010). Our present mechanistic understanding of mitochondria dynamics relies heavily on the relative frequency with which mitochondrial fission vs. fusion takes place in a cell. Frequent fission results in small mitochondria whereas a reduced fission frequency or an increased tendency to fuse leads to large mitochondria. Demonstrations of speculated stoichiometric relationships between the different mitochondria-associated proteins discovered so far greatly support this viewpoint but do not immediately suggest how two or more portions of an elongated mitochondrion might actually be forced apart.

The recent demonstration of tightly coupled dynamics and extensive membrane contacts between mitochondria and tubules of the endoplasmic reticulum (ER) in yeast and mammalian cells has been a major breakthrough in this context (Friedman et al., 2011; Friedman and Nunnari, 2014). Close associations between mitochondria and the ER have been known in plant cells for a long time (Gunning and Steer, 1986; Lichtscheidl and Url, 1990; Stickens and Verbelen, 1996) and have been used to support their biochemical relationship. However, the idea that the ER might be directly involved in the fission of elongated mitochondria into the predominant, punctate form in plant cells has not been explored to date. A major objective for our investigations was to assess a possible role of contiguous ER tubules and cisternae in mediating mitochondrial fission in plant cells.

The work on mitochondria-ER interactivity has focused largely on the phenomenon of mitochondrial fission and on identifying the protein complexes that aid inter-organelle interactions (Friedman et al., 2011). However, in a diversity of organisms including plants abnormally large mitochondria, called mega mitochondria and giant mitochondria have been observed under suboptimal conditions of growth and development including hypoxia, metabolic disorders, and chemical-induction (Tandler and Hoppel, 1986; Ramonell et al., 2001; Wakabayashi, 2002; Van Gestel and Verbelen, 2002). In addition, both animal (Grainger and James, 1969; Bowes and Gupta, 2008) and plant (Logan et al., 2004) cells exhibit sporadic thin extensions from and between mitochondria that have been called matrixules (Logan et al., 2004; Logan, 2006b; Scott et al., 2007). Elongated mitochondria also display a rather perplexing phenotype described as beads-on-a-string. It is unknown whether the ER plays a role in shaping all these different mitochondrial forms too. Whereas, earlier investigations have described the effect that specific conditions (Ramonell et al., 2001; Van Gestel and Verbelen, 2002) have on mitochondrial morphology, our study considers the entire range of mitochondrial pleomorphy in plant cells in relation to the organization and behavior of contiguous ER tubules and cisternae.

Our investigations involve time-lapse imaging of living cells expressing fluorescent proteins targeted to mitochondria (mito-GFP: Logan and Leaver, 2000; mito-mEosFP: Mathur et al., 2010) and the ER [ER-lumen retained RFP (RER); Sinclair et al., 2009]. Physiological manipulations on transgenic Arabidopsis plants have revealed that shifting plants between light and dark conditions of growth, changing cytosolic sugar levels through exogenous feeding of sucrose and maintaining seedlings transiently under hypoxia produce significant alterations in mitochondrial morphology. Our investigations involving a wide range of transient shapes including small spherical mitochondria, rings, and coiled, branched, and beaded tubules displayed by elongated mitochondria are reinforced through observations on abnormally long, fission-impaired mitochondria in the Arabidopsis *drp3a*/*adl2a* (Arimura et al., 2004; Logan et al., 2004) and *nmt1-2/elm1* (Logan et al., 2003; Feng et al., 2004; Arimura et al., 2008; Logan, 2010) mutants. In addition the *pah1pah2* double mutant in the phosphatidic acid phosphohydrolase1 and 2 that contains over-expanded ER cisternal membranes (Eastmond et al., 2010) was used to test possible association between mitochondrial form and ER. Our observations suggest a strong correlation between mitochondrial pleomorphy and the ER.

## Results

Earlier reports on transgenic tobacco and Arabidopsis plants, protoplasts, single cells, and detached organs (Logan and Leaver, 2000; Van Gestel and Verbelen, 2002; Logan, 2006a) that between them describe the complete range of mitochondrial pleomorphy in plants formed the basis for our optimization of procedures for 7–9 days old seedlings of stable transgenic *Arabidopsis thaliana* (Columbia ecotype) expressing mito-GFP. Subsequent visualization of punctate, elongated, and giant mitochondria and the ER simultaneously in double transgenic plants co-expressing mito-GFP and RFP-HDEL (RER; Sinclair et al., 2009) used our optimized methods.

### Cytosolic sugar levels and exposure to light affect mitochondrial size in plant cells

**Figure 1:**
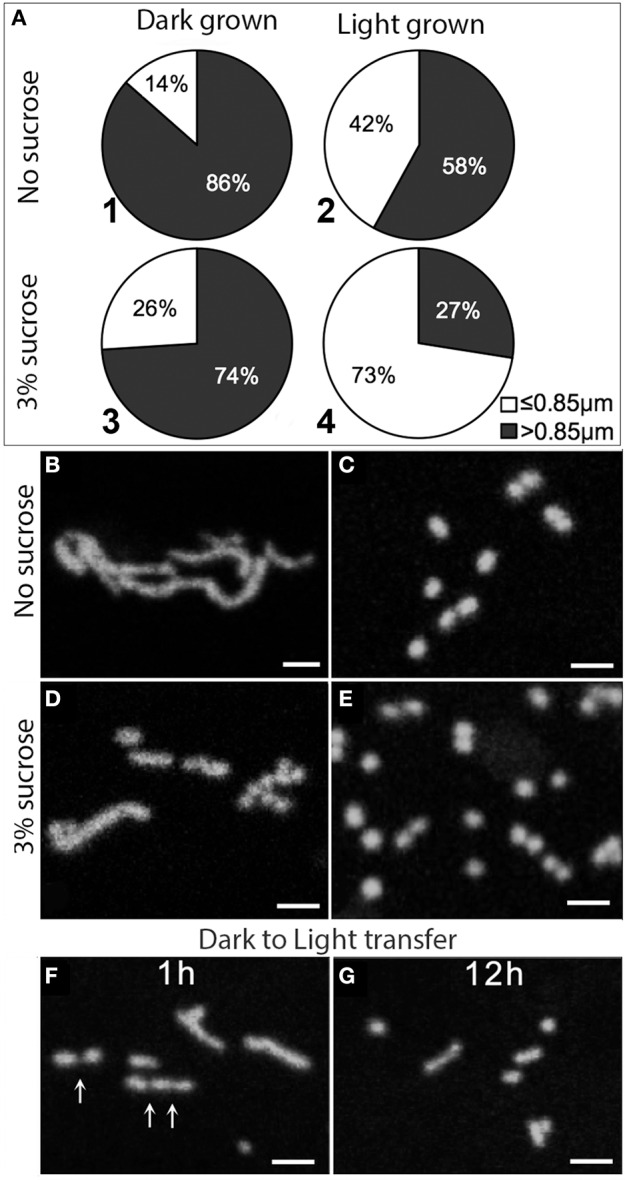
**Effects of light, dark and sugar on mitochondria length**. **(A–E)** Pie diagram showing the relative percentage of small (less than 0.85 μm) vs. elongated (longer than 0.85 μm) mitochondria in a cellular population for under different conditions. Data based on Arabidopsis mito-GFP transgenics grown for 7 days in light (70 μmol m^−2^ s^−1^) and dark on MS medium with no sucrose **(A1,A2,B,C)** and with 3% sucrose **(A3,A4,D,E)**. Mitochondria in seedlings grown in the dark were predominantly elongated **(B,D)** as compared to the punctate mitochondria in plants grown in light **(C,E)**. **(F,G)** Dark grown plants grown without sucrose were transferred to light (70 μmol m^−2^s^−1^) for 1 h **(F)** and 12 h **(G)** without sucrose. Putative fission sites on tubular mitochondria are indicated (arrows).

Photosynthesis in green plant cells causes cytosolic sugar levels to fluctuate considerably between cells and during the day and night cycle (Azcón-Bieto and Osmond, 1983; Azcón-Bieto et al., 1983). This was the rationale behind our using a more controlled, *in vitro*, exogenous sugar-feeding approach. Arabidopsis mito-GFP seeds were sown on MS medium with and without sucrose and kept in the dark to first assess if exogenous sugar in the MS medium actually altered cytosolic sugar levels in seedlings. Cytosolic sugar estimations (Figure 1; Supplementary Information) for 7 day old plants using a phenol-sulfuric acid colorimetric method (Buysse and Merckx, 1993) established that plants grown on MS medium with 3% sucrose had higher soluble sugar levels [10.14 ± 1.10 μg/mg of fresh weight (f.w.)] as compared to plants grown without sucrose in the medium (1.92 ± 0.77 μg/mg f.w.).

Confocal laser scanning microscopy of hypoctyl cells of mito-GFP revealed that although all cells had a mixture of punctate (≤0.85 μm long) and elongated (>0.85 μm long) mitochondria, the relative frequency between these two broad categories varied. Eighty-six percent of mitochondria were elongated when plants were grown in the dark without sucrose (Figures 1A1,B), which decreased to 74% for plants grown in the dark with 3% sucrose (Figures 1A3,D). The observations on dark grown plants had to be taken quickly since rapid fission of elongated mitochondria occurred within minutes and changed the relative number of small vs. elongated mitochondria in a cell. Whereas, 58% of mitochondria were elongated in plants grown in light without sucrose (Figures 1A2,C), only 27% were elongated in plants grown in the light with sucrose. Furthermore, the light and sucrose grown plants conversely had the highest proportion of small, round mitochondria (Figures 1A4,E).

The effect of light on mitochondrial fission was confirmed in a separate experiment by taking plants grown in the dark without sugar with clearly elongated mitochondria and transferring them to 70 μmol m^−2^s^−1^ light for 1 and 12 h (Figures 1F,G). In both cases the predominant mitochondrial size approached ≤ 0.85 μm.

We concluded that darkness and low sugar levels favor an elongated mitochondrial morphology whereas high light with and without high cytosolic sugar drives mitochondrial fission to create the punctate mitochondria considered typical of plant cells. Having observed that elongated mitochondria can form in normal plant cells under sugar-depleted conditions, we turned to other ways of obtaining a change in mitochondrial form.

### Oxygen limited conditions induce mitochondrial fusion and alter mitochondrial form

**Figure 2:**
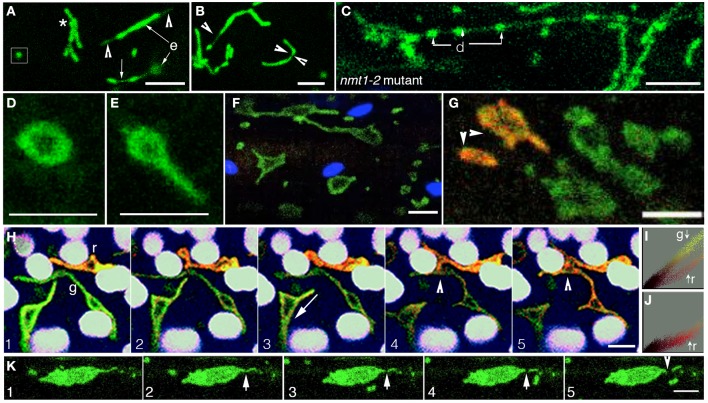
**The range of mitochondrial forms obtained in Arabidopsis plants under different conditions**. **(A)** Mitochondria in dark grown hypocotyl cells could appear punctate (box), clustered (^*^), or elongated (e). An elongated mitochondrion could exhibit terminal (arrowheads) or medial matrixules (small arrow). The medial matrixule was often observed just before the fission of an elongated mitochondrion. **(B)** Elongated mitochondria displayed shape contortions that eventually led to mitochondrial fission. Thin, stretched out regions, as shown in an elongated mitochondrion (single arrowhead) and a possible break-point shown in a sickle-shaped mitochondrion (facing arrowheads) were commonly observed. **(C)** Elongated mitochondria from dark-grown plants often exhibited a beads-on-a-string form consisting of dilated (d) and narrow intervening regions in the nmt1-2 mutant. Whereas, in elongated mitochondria in the wild type this form often preceded fission, it persisted in the fission-impaired *adl2a* and *nmt1-2* mutants. **(D,E)** Single, expanded torus-like mitochondrial forms were observed under oxygen-limited conditions. As suggested by the extension in **(E)** this shape is not fixed and changes over time. **(F)** Under prolonged hypoxia enlarged mitochondria expanded into flattened discs and sheet-like forms. **(G)** Hypoxia in a plant expressing mitochondrial targeted mEosFP resulted in expanded mitochondria, some of which (arrowheads) were photo-converted from green to orange-red. **(H)** A time-lapse image series showed the interaction (frames 1–4) and eventual fusion (frame 5-arrowhead) of a green (g) and a photo-converted, red (r) mitochondrion to produce an even larger giant mitochondrion (frame 5) with an intermediate orange fluorescence. **(I,J)** A color distribution map of frame1 compared to frame 5 in **(H)** provided an estimate of redistribution of mitochondrial matrix through the prominent color mixing that occurred upon fusion of the green non-photo-converted with the red, photo-converted mitochondrion. **(K)** A time-lapse image sequence obtained after irradiating a giant mitochondrion formed under low oxygen conditions **(1)** with bright light for 6 min initiated a break point (arrowhead in **2**), progressive separation of a small fragment **(3,4)** and its eventual moving away from the large mitochondrion. Size bars = 5 μm.

In agreement with earlier reports (Van Gestel and Verbelen, 2002; Logan, 2006a) seedlings with approximately 1.5–2 μm long mitochondria, when kept between a glass slide and coverslip for more than 30 min showed an increase in mitochondrial lengths of up to 5–8 μm (Figures 2A,B). Mitochondrial elongation took place irrespective of whether the seedlings were grown in the dark or light, with or without sucrose. The considerably elongated mitochondria displayed normal motility rates of 1.5 ± 0.4 μms^−1^ and morphed continuously, coiling, and forming branched and reticulate shapes (Figure 2B). Time-lapse imaging of tubular mitochondria showed sporadic dilations and constrictions forming along their length resulting in a beads-on-a-string appearance (Figure 2C). Exposing the plants to 800 ± 50 μmol m^−2^s^−1^ light for approximately 30 s using the bright-field illumination on our upright epi-fluorescence microscope led to the rapid fission of such mitochondria into smaller units. In nearly all cases the beads-on-a-string form preceded a fission event with the actual break occurring at the narrow neck region (Figures 2A,B). Occasionally, single mitochondria displayed thin transient extensions that have been called matrixules (Scott et al., 2007; Figure 2A).

Under the conditions described above it was difficult to observe elongated mitochondria in mito-GFP for more than a few minutes without their either undergoing fission or progressing into relatively inactive enlarged mitochondria. In order to reliably observe the numerous shapes that elongated mitochondria adopt transiently, we used the fission impaired *nmt1-2/elm1-1* mutant (Logan et al., 2003; Arimura et al., 2008; Logan, 2010). Eight to ten days old mutant seedlings grown in a 16 h light/8 h dark cycle were used and displayed 8 ± 3 μm long mitochondria in cotyledon cells. Interestingly nearly 70% of the mitochondria in hypocotyl cells initially appeared normal. However, within 2 min mitochondria commenced fusing and elongating rapidly. Similarly in cotyledon cells of the *nmt1-2* mutant, there was an increase in mitochondrial length up to 20–25 μm and the formation of tubular, branched networks.

It was concluded that while light promoted fission mitochondrial fusion was favored under a low oxygen environment. This led to mitochondrial elongation in both wild type and *nmt1-2* and *drp3a* mutants. Contrary to the light-triggered fission of elongated mitochondria in wild type plants, the greatly elongated mitochondria in *nmt1-2/elm1-1* and *drp3a* mutants continued morphing for longer periods. Mitochondria in the mutants also displayed a high frequency of matrixule formation and the beads-on-a-string phenotype (Figure 2C; *nmt1-2* mutant).

However, if mito-GFP and *nmt1-2/elm1-1* seedlings were left immersed in water for more than 45 min (maximum time assessed was 3 h) mitochondrial motility decreased from 1.5 ± 0.4 μms^−1^ to approximately 0.5 ± 0.2 μms^−1^ or displayed an oscillatory motion. All mitochondria exhibited isotropic expansion around this time (Figures 2D–F). Average numbers and sizes could not be obtained due to the wide variability displayed even within a single cell. In both wild type and mutant plants, some mitochondria remained as relatively immobile expanded discs (Figures 2D,E) while others enlarged into flat, expanded, irregular shapes to form giant mitochondria ranging in length from 10 to 16 μm (Figure 2F).

Earlier studies have suggested that giant mitochondria form due to hypoxia (Ramonell et al., 2001; Van Gestel and Verbelen, 2002; Logan, 2006a). This observation was confirmed in wild type seedlings by overlaying seedlings on the slide with mineral oil, a standard microbiological procedure for creating anoxic conditions (Edwards et al., 1947; Jacobson et al., 2004). Since the polygonal shapes adopted by giant mitochondria (Figure 2F) appeared quite different from the annulus-like, iso-tropically expanded mitochondria (Figure 2D) we investigated whether giant mitochondria formation involves fusion.

### Formation of giant mitochondria involves fusion of several expanded mitochondria

A stable transgenic Arabidopsis line expressing a mitochondrial-targeted green to red photo-convertible monomeric Eos fluorescent protein (mito-mEosFP; Mathur et al., 2010) was used for these experiments (Figure 2G). Seedlings were immersed in water on a slide and single mitochondria were observed progressing through the elongated phase into the large, expanded forms. Single flattened mitochondria within different clusters were photo-converted (Figure 2G; arrowheads). Close interactions between photo-converted (red) and non-photo-converted mitochondria (green) over several minutes resulted in their fusion and the formation of a giant mitochondrion (Figure 2H; Supplementary Movie 1) with an intermediate, yellow-orange color (Figures 2H5,I,J). Whereas, observations of more than 20 such events clearly established that the formation of giant mitochondria involves the fusion of already expanded mitochondria, the initial distance between the fusion pair and the time taken for fusion were very variable and made it difficult to predict the degree of interactivity required for the event. We wondered whether the giant mitochondria represented a terminal phase in mitochondrial morphology or could be made to undergo fission again. This was investigated next.

Whereas, tubular mitochondria undergo rapid fission upon exposure to high light (800 ± 50 μmol m^−2^s^−1^) for less than a minute achieving a similar degree of fission in giant mitochondria required a longer exposure time of more than 10 min. However, as shown (Figure 2K) giant mitochondria do undergo fission and produce small mitochondria with irregular forms.

Having observed the range of mitochondrial pleomorphy and optimized conditions for obtaining mitochondrial sizes ranging from punctate (≤0.85 μm long) to giant (>8 μm long) mitochondria in 7 day old Arabidopsis seedlings we undertook the simultaneous visualization of mitochondria and the ER to assess their possible relationship.

### Mitochondrial fission correlates with the reorganization of ER tubules

**Figure 3:**
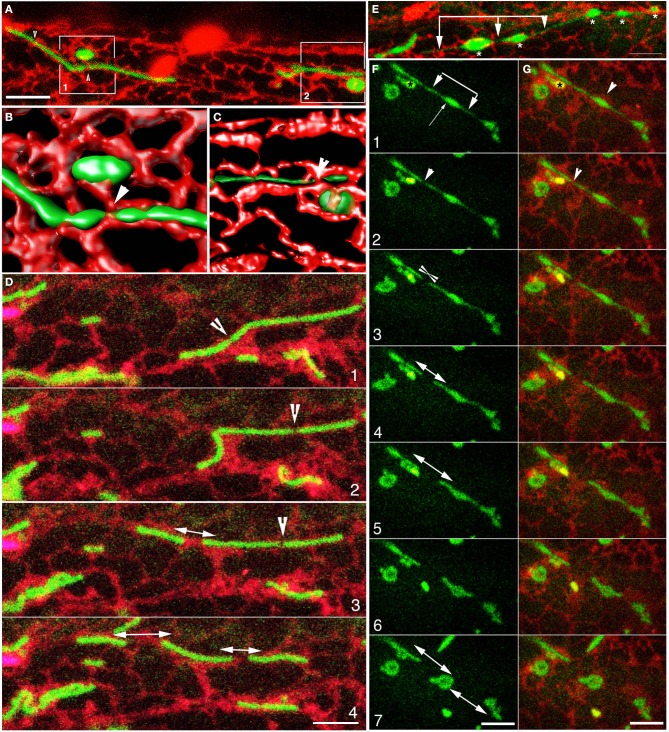
**Contortions and fission of elongated mitochondria occur through physical interactions with the ER mesh**. **(A)** A collapsed confocal image stack shows two elongated and two swollen, oval mitochondria embedded in the ER. Arrowheads indicate ER bands that encircle the tubular mitochondria. **(B)** Iso-surface volume rendering of boxed-in region 1 in **(A)** depicts the 3-dimensionality of the mitochondrion-ER spatial relation. An oval mitochondrion is located within an ER-cup while a tubular mitochondria appears threaded between ER polygons. Note the constrictions in the elongated mitochondrion due to encircling ER bands (e.g., arrowhead). **(C)** 3-D volume rendering of boxed-in region 2 in **(A)** shows a tubular mitochondrion enmeshed in the channel created by contiguous ER polygons of variable diameter. Increasing the transparency of the green channel has allowed an appreciation of the ER tubules lying behind the mitochondria. **(D)** Four snapshots selected from a time-lapse series (Supplementary Movie 2) show an elongated mitochondrion (panel 1) with a putative fission site (arrowhead) contorting (panel 2) before breaking (panel 3). The distance between the longer portion of the mitochondria and the broken off segment widens in panels 3 and 4 while another break appears (arrowhead in panel 3) and complete fission of one elongated mitochondria into three is achieved in panel 4. Note that the contiguous ER also stretches and rearranges during this process. **(E)** An image suggesting that different portions of mitochondria can become enmeshed within different ER polygons and thus get stretched during the dynamic reorganization of the ER reorganizes. The stretching creates dilations (^*^) and narrow regions (arrows) in the elongated mitochondrion suggesting the beads-on-a-string form. The terminal, thin regions may also be interpreted as matrixules. **(F,G)** Snapshots acquired from a time-lapse image sequence show the stretching of an elongated mitochondrion and the formation of thin regions (arrowheads in **F**1) suggesting matrixules. Note that the dilated region (**G**1; arrowhead) locates to enlarged ER cistern. Panels **2–5** show fission and expanding ER cisternae in G that increase the gap between the different mitochondrial segments. Panels **5–7** suggest a snap-back phenomenon where the mitochondrial segments curl up on themselves as the neighboring ER completes their separation. A single yellow fluorescent peroxisome (^*^in panel 1) was also observed in this image series. Size bars = 5 μm.

Simultaneous imaging of mitochondria and the ER in Arabidopsis transgenic seedlings co-expressing mito-GFP and RER revealed that mitochondria were nestled within polygons created by ER tubules. Time-lapse images showed further that the seemingly erratic movement of mitochondria correlated with the motility and organization patterns of ER tubules (Supplementary Movie 2). Immersion of seedlings in water under a coverslip for 30–45 min allowed mitochondria to elongate without a noticeable change in ER motility. Subsequent exposure to bright light for 30 s triggered mitochondrial fission. Through highly reproducible time-lapse series taken in over 80 different cells from 10 plants and from iso-surface volume rendering of confocal image stacks it became apparent that single elongated mitochondria were encircled by multiple ER tubules at several positions (Figures 3A–C; Supplementary Movie 2). The position of these ER bands around mitochondria often changed during the reorganization of the dynamic ER mesh. Changes in the organization of ER tubules and the consequent narrowing or broadening of ER polygons resulted in the stretching and twisting of enmeshed mitochondria. Whereas, an average time for a fission event could not be estimated due to the rather asynchronous interactions of mitochondria and the ER, a medial narrowing or constriction of the mitochondrion preceded every such event (Figures 3D,E; Supplementary Movie 3). This narrowing usually formed the point of fission (Figures 3F,G). A certain amount of force to separate the mitochondrial fragments was indicated in the way that the two portions of the mitochondrion and the ER pulled away into opposite directions after a fission event (Figures 3F,G). The pulling force often created a thin region between two fragments, very much like a medial matrixule, prior to their actual separation (Figures 3F,G).

Since the formation of matrixules (Logan et al., 2004; Logan, 2006b; Scott et al., 2007) and the beads-on-a-string form are interesting transient phenomenon the role of the ER in such occurrences was investigated next.

### Transient matrixules and the “beads-on-a-string” form are created when elongated mitochondria navigate through the ER mesh

Our assessment of the pleomorphy of elongated mitochondria in mito-GFP plants as well as the *nmt1-2/elm1-1* mutant revealed a wide range of transient shapes including polygons, rings, coiling, branched, and beaded tubules (Figure 2). As mentioned earlier, in wild type plants the mitochondrial contortions invariably led to stretching and breaking at weak points. However, elongated mitochondria such as those found in the mitochondrial fission-impaired *nmt1-2/elm1-1* plants co-expressing mito-GFP/Mt-GFP and RER appeared to be threaded through the different-sized polygons making up the ER mesh. During their trajectory through the mesh the elongated mitochondria sporadically displayed thin regions and dilated areas that conveyed an impression of beads-on-a-string (Supplementary Movie 4). We obtained a similar impression upon observing mitochondria stained with MitoTracker in the *adl2/drp3a* mutant (Arimura et al., 2004; Logan et al., 2004), which is also impaired in fission.

The observation that mitochondrial fission is aided by the activity of neighboring ER tubules suggested that the variation in the size of mitochondria might be correlated with the size of ER polygons in a cell. This was investigated next.

### Mitochondrial length correlates with the size of surrounding ER polygons

**Figure 4:**
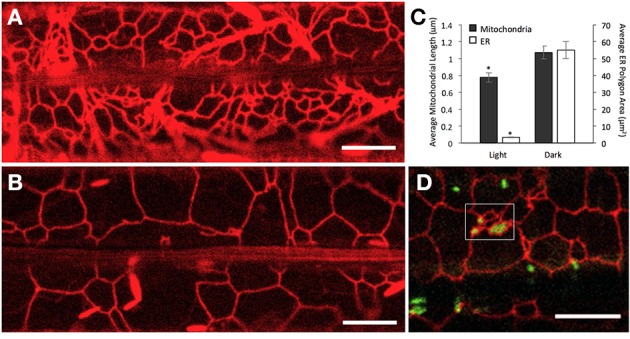
**Average size of ER polygons and mitochondria correlates under light and dark growth conditions**. **(A,B)** Representative images from 8 day old plants of RER Arabidopsis transgenics grown in the light (164 μmol m^−2^s^−1^) **(A)** and complete darkness **(B)** show the visible difference in the size of ER polygons. **(C)** A comparison of mitochondria and ER size from plants grown in the light and dark. Mitochondria from mito-GFP plants (*n* = 200 mitochondria per treatment) were significantly longer when plants were grown in the dark than in the light (1.07 ± 0.34 and 0.78 ± 0.15 μm, dark and light, respectively; *p* < 0.01). The average ER polygon area was also significantly larger in dark grown plants than those grown in the dark (55.07 ± 39.44 and 3.27 ± 2.05 μm, dark and light respectively; *n* = 120 ER polygons per treatment; *p* < 0.01). Standard error bars are shown. **(D)** “Corrals,” regions with small ER polygons formed between large ER polygons (boxed in area) were observed in dark grown plants. Mitochondria enmeshed in these regions were small while mitochondria elsewhere in the cell appeared elongated. The number of small ER polygons increased upon exposure to light and coincided with the increase in population of small mitochondria. Scale bars in **(A,B,D)** = 10 μm.

Our earlier observations had established that while mitochondria in plants grown under light are small, there is a significant increase in the sub-population of elongated mitochondria in the dark (Figure 1). A possible correlation with the ER was sought by comparing mito-GFP-RER seedlings grown under dark and light conditions. In comparison to the meshwork of small ER polygons under light-growth conditions (Figure 4A), the polygons were significantly larger in dark grown seedlings (Figures 4B,C). In general, the increased ER-polygon size correlated well with increased mitochondrial length in the dark (Figure 4C). Therefore, it was perplexing to find some small mitochondria too in dark grown plants. The reason for this apparent discrepancy was traced to the presence of numerous small ER polygons that are formed between large ER-polygons (Figure 4D). Based on our observation of numerous small mitochondria trapped in these small-ER-polygon enriched regions it appeared that elongated mitochondria enmeshed within such pockets, named “corrals,” broke up to form small mitochondria. Time-lapse imaging of dark grown plants following their exposure to light revealed that the formation of ER corrals increased over time so that after a few hours in light, the ER network comprised predominantly of small polygons. Notably there was a concomitant increase in the population of small mitochondria within the cell. Thus, our observations clearly indicated that the length of a mitochondrion in wild type plants depends upon the size of contiguous ER polygons and small polygons correlate with increased mitochondrial fission.

We concluded that all transient forms exhibited by elongated mitochondria were in response to their physical interactions with neighboring ER and frequently led to fission. However, these contorted forms differed considerably from the expanded, sheet-like mitochondria observed under hypoxia. We asked whether the mitochondria-ER relationship continues under oxygen limited conditions and investigated this next.

### Hypoxia-induced mitochondrial forms correlate with expanded ER cisternae and reduced polygon formation by ER tubules

**Figure 5:**
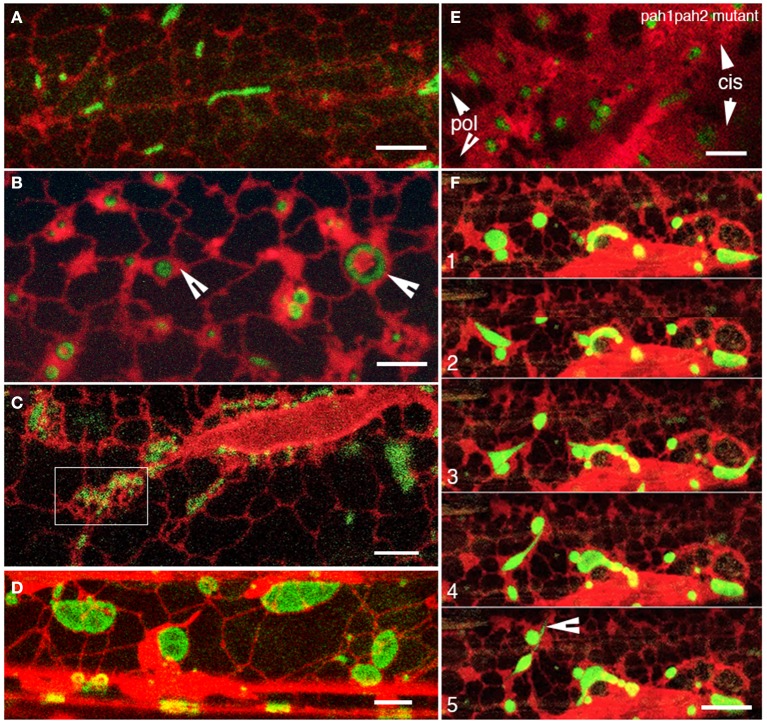
**Reduced ER dynamics promote mitochondrial fusion to form giant mitochondria**. **(A)** Representative view of the ER organization and assorted mitochondrial size in normal, light-grown hypocotyl cell. **(B)** Representative view of expanded ER cisternae surrounding almost isotropically expanded mitochondria after 1 h under oxygen-limited conditions. **(C)** Mitochondrial clusters in ER corrals (e.g., boxed in region) surrounded by relatively large ER polygons were observed in dark-grown plants. **(D)** A representative image from a dark plant similar to **(C)** after an hour of oxygen limited conditions shows flat, giant mitochondria that appear to have formed through the fusion of mitochondria caught up in ER corrals. Note that there are no unduly expanded cisternae. **(E)** Representative image showing rather diffuse, enlarged mitochondria embedded in greatly expanded ER cisternae (cis) and not very well defined ER polygons (pol) in the *pah1pah2* double mutant. **(F)** Snapshots from a time-lapse series of a cell with expanded mitochondria and ER cisternae exposed to light showed the resumption of dynamic behavior for both organelles. Mitochondrial blobs started extending and stretching and eventually breaking up as contiguous ER polygons got reorganized. A matrixule–like projection was extended from an expanded mitochondrion resuming dynamic behavior (arrowhead in panel F5). Size bars **(A–E)** = 5 μm; **(F)** = 10 μm.

As optimized earlier, the immersion of seedlings in water for about 45 min to an hour resulted in a general expansion of mitochondria. For light grown seedlings the simultaneous visualization of the ER and mitochondria at this stage showed a gradual reduction in the motility of both organelles with concomitant expansion of ER cisternae and single mitochondria (Figure 5A vs. Figure 5B; arrowheads in Figure 5B). For dark grown plants with small mitochondria clustered in ER corrals (Figure 5C-box) the expanded ER cisternae did not become as apparent as in light-grown plants. However, ER motility and the rearrangement of ER polygons did slow down and enlarged, flattened mitochondria became evident within the same duration as light grown plants (Figure 5D). Time-lapse observations suggested that reduced ER dynamics increased interaction time between mitochondria and promoted their fusion. These observations were tested in the *nmt1-2/elm1-1* mutant transformed with RER. While the mutant has characteristically elongated mitochondria we were able to observe punctate mitochondria, by growing plants on 3% sugar containing MS medium and exposing them to light for 6 h before subjecting them to hypoxia. Punctate mitochondria clustered rapidly, fused and converted into long tubules within 15 min (Supplementary Movie 5). While the fusion in *nmt1-2/elm1-1* mutant took place quickly and the ER appeared slightly diffuse and gave the impression of having expanded cisternae we used another mutant to reinforce this point. An Arabidopsis double mutant *pah1pah2* in the phosphatidic acid phosphohydrolase1 and 2 contains over-expanded ER cisternal membranes (Eastmond et al., 2010) and was used to address this question. RER and mito-GFP dual probe expression in the *pah1pah2* double mutant background showed expanded mitochondria with a diffuse outline in regions with large cisternae (Figure 5E).

In order to complete our investigation on ER involvement in mitochondrial pleomorphy we considered that reinvigorating the ER into producing and rearranging tubules should break up the giant mitochondria. Indeed exposing seedlings with a rather moribund ER and giant mitochondria to light for up to 10 min resulted in resumption of ER tubule extension and polygon formation. In over 25 observations this reactivation coincided with changes in the shape of giant mitochondria and their fission into smaller mitochondrial units. Similar patterns of dilations and thinning and stretching as described earlier were observed (Figure 5F; Supplementary Movie 6).

## Discussion

### A high energy status creates and maintains the predominantly small mitochondria in green plant cells

Mitochondria are routinely described as dynamic, pleomorphic organelles (Cavers, 1914; Lewis and Lewis, 1915; Bereiter-Hahn and Vöth, 1994; Nunnari et al., 1997; Logan and Leaver, 2000; Youle and van der Bliek, 2012; Friedman and Nunnari, 2014). Whereas, elongated mitochondria have been described in green algae (McFadden and Wetherbee, 1982 and references therein), characean internodal cells (Foissner, 2004), in leaves of Ficus (Duckett and Toth, 1977) and Arabidopsis (Ramonell et al., 2001), and in tobacco cells (Stickens and Verbelen, 1996; Van Gestel and Verbelen, 2002) several years of live-imaging using vital dyes and mitochondria-targeted fluorescent proteins have led to the general view that their predominant form is small and punctate in most green plants (Matzke and Matzke, 1986; Köhler et al., 1997; Logan and Leaver, 2000). However, the basis for the formation and maintenance of the discrete, punctate mitochondrial form in plants is unclear and therefore formed the focus of our investigations.

Our observations reveal that significant changes in mitochondrial size occur in response to alterations in the cytosolic sugar levels in plant cells. We demonstrate that whereas cells in plants kept in the dark and starved of sugar exhibit elongated mitochondria, sugar-replete cells predominantly exhibit small mitochondria. These observations agree with an energy dependent internal arrangement of mitochondrial cristae that is believed to underlie mitochondrial dynamics (Bereiter-Hahn and Vöth, 1994; Van der Klei et al., 1994). In green, photosynthesizing plants there are considerable fluctuations in the sugar status of a plant cell between the day and night periods (Azcón-Bieto and Osmond, 1983; Azcón-Bieto et al., 1983; Taiz et al., 2015). In addition we found that exposure to light also results in small mitochondria. While light is the major driver of photosynthesis it is also responsible for changes in the redox status of a plant cell (Douce, 1985; Siedow and Umbach, 1995; Noctor et al., 2007). In plants chloroplasts are major contributors to subcellular reactive oxygen species (ROS). At day break the photo-conversion of proto-chlorophyllide, enriched in chloroplasts during the dark period, into chlorophyll generates different ROS (Meskauskiene et al., 2001) and might trigger rapid mitochondrial fission. Here we have not investigated ROS involvement in mitochondrial fission in depth but subcellular ROS production might also be augmented through reactions in the mitochondrial electron transport chain (ETC) (Logan (2006a). Observations on hyperglycemic animal cells with small, punctate mitochondria similar in appearance to those found in the cells of green plants strongly suggest that a similar sugar-ROS link might operate in green autotrophic plants. Clearly the high-energy state of plant cells during the day favors the formation and maintenance of a population of small-sized mitochondria while a relatively lower energy status at night makes mitochondria more elongated.

### The ER has a role in making elongated mitochondria small

Following our understanding of cellular energy status as the major factor affecting mitochondrial size we investigated the mechanism through which elongated mitochondria broke into smaller units. Active fission of elongated mitochondria was indicated. Proteins such as Fis1p/hFis1/FIS1 and Dnm1/Drp1/DRP3 are firmly implicated in maintaining the fission-fusion cycle in different organisms including plants (Hoppins et al., 2007; Logan, 2010; Rafelski, 2013). In addition ER tubules have been identified as key players that exercise pulling force to promote mitochondrial splitting (Friedman et al., 2011; Lackner et al., 2013; Friedman and Nunnari, 2014). While ER-mitochondrion co-visualization has been achieved for dividing tobacco protoplasts and expanding and regenerating cells (Stickens and Verbelen, 1996) to the best of our knowledge, no investigations have approached the possible involvement of the ER in mediating mitochondrial fission and pleomorphy in plants. Our observations show that dark grown plants have large ER polygons compared to plants grown in light. This observation correlates well with the presence of elongated mitochondria in the dark and small mitochondria in light. Further, using time-lapse imaging we found that in response to light the tubules making up the ER mesh rearrange into smaller sized-polygons and their remodeling increases the frequency of mitochondrial fission. Our observations concur with findings on ER-mitochondrial co-operation in animal and yeast cells (Friedman et al., 2011; Kornmann, 2014). However, our work has gone one step further by investigating the role of the ER in mitochondrial fusion.

### The ER has a role in mitochondrial fusion

The mechanism for mitochondrial fusion in plants remains unknown as many of the proteins involved in this process in animal and yeast cells appear to have no clear homologs in plants (Zhang and Chan, 2007). A study by Sheahan et al. (2005) on mitochondrial fusion in plants utilized protoplasts and several inhibitors to conclude that mitochondrial elongation results from massive mitochondrial fusion and requires IMM electric potential, cytoplasmic protein synthesis, microtubules, and functional kinesin but not ATP or actin filaments. Interestingly, inhibitors used to disrupt actin filaments stopped all mitochondrial movement except Brownian oscillations but did not hinder fusion or fission whereas the myosin inhibitors 2,3-butanedione monoxime (BDM) and N-ethylmaleimide (NEM) affected both mitochondrial motility and mitochondrial fusion negatively (Sheahan et al., 2005). These findings on myosin inhibitors reinforce our own observations since these chemicals directly affect ER dynamics (Liebe and Menzel, 1995; Ostap, 2002). Our findings that mitochondria elongate in the dark and on low sugar levels suggested that fusion rather than fission was being favored under these conditions. A correlation was found in concomitant decrease in ER motility and enlarged polygon size. Further support for increased fusion was obtained under increasing hypoxia when both mitochondrial motility and ER polygon rearrangement slowed down considerably. In addition, the ratio between tubular ER and flattened cisternae changed and most single mitochondria started exhibiting a characteristic isotropic swelling. Mitochondria often became clustered between expanded ER cisternae and over time fused to form giant mitochondria. While we have concluded that a moribund state of the ER might be responsible for creating giant mitochondria, it is equally possible that the low energy status of mitochondria under hypoxia causes the surrounding ER to flatten out first and then subsequently affects the mitochondria as well. Our observations match the commonly observed formation of giant or mega mitochondria in response to oxygen deprivation and inhibition of respiration (Tandler and Hoppel, 1986; Bereiter-Hahn and Vöth, 1994 and references therein; Karbowski et al., 1999; Teranishi et al., 1999; Vartapetian et al., 2003). In aerobic organisms, the efficient generation of ATP during oxidative phosphorylation requires oxygen (O_2_). Limiting the supply of O_2_ thereby interferes with the efficiency of mitochondrial ATP generation, ultimately limiting the cellular energy status. Similar observations that plant cells maintained under a cover-slip in water start exhibiting elongated/expanded mitochondria after about 30 min have been made by Van Gestel and Verbelen (2002) and Logan (2006a). Therefore, an important consideration to be kept in mind while viewing mitochondria is that their shapes may change during and due to the process of imaging itself.

### Contorted mitochondrial forms result from close alignment with the ER

Another manifestation of the ER-mitochondria cooperation observed by us was the frequent morphing of elongated mitochondria to form apparent networks, sinous forms, loops, circles, a beads-on-a-string phenotype, and the formation of terminal and median matrixules that are characteristic of mitochondria in living plant cells. Conventionally these shapes have been attributed to rearrangements of internal mitochondrial membranes (Bereiter-Hahn and Vöth, 1994). However, Logan (2006a) has questioned this interpretation based on intrinsic factors only and pointed out that the activities of molecular motors and the cytoskeleton should also be considered. While this work has not investigated the involvement of motor proteins and the cytoskeleton *per se* our observations strongly suggest that all mitochondrial contortions result from their alignment with contiguous ER tubules. However, the dynamic behavior of the ER and mitochondria is intimately associated with both the actin cytoskeleton and myosin motors (Van Gestel et al., 2002; Avisar et al., 2008; Ueda et al., 2010; Peremyslov et al., 2012; Joensuu et al., 2014). Thus, there is a possibility that actin-myosin dependent mitochondrial dynamics also influence the behavior of contiguous ER. A general organelle interactivity model where each subcellular compartment influences the other is being actively investigated by us.

### ER-mitochondrial association explains matrixules and the beads-on-a-string-phenotype

The use of *nmt1-2/elm1-1* and *adl2a/drp3a* mutants allowed us to explore the behavior of elongated mitochondria in more detail and revealed a key role for the ER in creating two interesting morphologies described in mitochondrial literature on plants. Several elongated mitochondria adopt a transient beads-on-a-string shape comprising of narrow and swollen areas while others display matrixules (Logan et al., 2004; Logan, 2006b; Scott et al., 2007). The matrixule has been described as a thin, several micro-meters long protuberance that extends from individual mitochondrion (Logan et al., 2004). According to Logan et al. (2004) matrixules are rarely observed in wild type plants but observed frequently in the *adl2a/drp3A* mutants and might form as a mitochondrion is being pushed through a constrictive collar such as a mitochondrial division ring comprising of DRP3/ADL2 and other proteins (Logan, 2006b). Matrixules may also have a terminal or a medial location (Logan: Supplementary Movie 4: http://www.plantmitochondria.net/Plant_Mitochondria/Movies.html). Whereas, observations of matrixules were made primarily in the *adl2a/drp3A* mutants (Logan et al., 2004), the *nmt1-2/elm1-1* mutant also provided succinct examples of the beads-on-a-string morphology (Arimura et al., 2008). Our observations suggest that both matrixule formation and the beads-on-a-string phenotype are not limited to the two mutants but can be observed in all elongated mitochondria. Further we demonstrate that both transient forms result as mitochondria move through the continuously rearranging ER mesh. We agree with Logan's (2006b) view that matrixules form as a mitochondria passes through a constrictive collar and based on our time-lapse images and 3D volume renditions (Figure 3) submit that the collar also includes ER tubules and not just proteins implicated in mitochondrial fission. The resultant view also suggests that as a flexible, elongated mitochondrion encounters the different-sized openings in the ER mesh during its motor-driven, ER-aided motility, the tubule becomes squeezed in some places and dilated in others to produce the beads-on-a-string form. Our view does not reduce the importance of the DRP3A and NMT1/ELM1 protein localizations (Arimura et al., 2004, 2008; Logan et al., 2004) but points to the involvement of the ER-membrane scaffolding for the mitochondrial fission complex.

Questions that remain unanswered in our study relate to why and how a certain degree of alignment or attachment between mitochondria and the ER is created. The presence of membrane contact sites (MCS) between the two organelles and protein complexes localized to these MCS have been described in other organisms (Friedman et al., 2011; Prinz, 2014) and can readily explain such coordinated behavior. While proteins with similar activity and localization patterns have yet to be identified in plants our observations certainly lay down the basis for such investigations. In a more general eukaryotic cell scenario our live-imaging based view points to the ER mesh as a physical barrier with which mitochondria and possibly other organelles interact as they move around the cell. As demonstrated by us these physical interactions mold organelle morphology. The involvement of cytoskeletal elements and motor proteins during the interactive processes poses interesting questions that require further work.

## Materials and methods

### Transgenic lines and plant growth conditions

*Arabidopsis thaliana* transgenic plants expressing a mitochondrial targeting signal sequence of the beta-ATPase subunit fused to Green Fluorescent Protein (mito-GFP; Logan and Leaver, 2000) or to photo-convertible mEosFP (Mathur et al., 2010) were used for observing mitochondrial morphology and dynamics. The *Agrobacterium* floral dip method (Clough and Bent, 1998) was used transform both mito-GFP and *nmt1-2/elm1-1* (At5g22350; Arimura et al., 2008; Logan, 2010) to create double transgenics lines expressing both RFP-HDEL (RER; Sinclair et al., 2009) and mito-GFP. The *adl2a/drp3a/apm1-1* mutant used has been described earlier (Arimura et al., 2004; Logan et al., 2004; Mano et al., 2004). The *pah1pah2* mutant (Eastmond et al., 2010) was transformed separately with mito-GFP and RER constructs and resultant transgenic plants were crossed to obtain the double transgenics in the double mutant background. Seeds were sterilized using commercial bleach, and unless stated otherwise, plated on Murashige and Skoog (1962) medium (MS) with and without 3% sucrose, stratified at 4°C for 2 days and then grown at 21°C for 7 days at 70 μmol m^−2^s^−1^ under a 16/8 h light dark cycle.

To determine the effects of sugar on mitochondrial morphology and dynamics, seedlings were grown on MS medium with 0 and 3% sucrose. For light-dark comparisons, seedlings on MS medium with and without sucrose were grown in light (70 μmol m^−2^ s^−1^, unless stated otherwise) and in complete darkness, while maintaining all other growth conditions as described earlier. Plants grown in the dark without sucrose were transferred to light (70 μmol m^−2^ s^−1^) for 1 and 12 h. Unless stated otherwise, all observations of mitochondrial and ER morphology were made in cells within the mid region of the hypocotyl. Cells around the vasculature were not considered as they usually exhibit longer mitochondria than epidermal and cortical cells.

### Sugar quantification

The phenol-sulfuric acid colorimetric method for quantifying the total soluble sugar of plant tissue described by Buysse and Merckx (1993) was implemented to determine the relative levels of sugar of plants grown in the light and dark. Plants were grown in the light (155 μmol m^−2^s^−1^) or dark for 7 days on MS medium with or without 3% sucrose. Fifty plants were harvested from each treatment either in light 1.5 h into the light cycle or in a room in near darkness. The plants were placed into pre-weighed 2 mL microfuge tubes and immediately frozen in liquid nitrogen and the tubes weighed again. Tissue fresh-weight was calculated by subtracting the post-harvest weight from the pre-weighed weight of the tube.

#### Sample preparation

The tissue was ground using magnetic beads and a bead beater (Retsch MM301) for 2 min at a frequency of 30.01/s and placed on ice immediately. After removing the magnetic beads, 500 μL of 80% ethanol was added to the tissue and vortexed to homogenize the tissue in solution. The tubes were centrifuged at 14,000 rpm for 5 min at 4°C. The supernatant was transferred to a new tube on ice. This elution in ethanol was repeated two more times. After the final elution, tubes were centrifuged one more time to remove residual tissue that could hamper quantification.

#### Quantification

A stock of 100 μg/mL of glucose in 80% ethanol was diluted to 10–90 μg/mL. These were used to create a standard line. Five hundred microliters of the standard or sugar elution was added to a cuvette. To each cuvette, 500 μL of 28% phenol (w/w) was added. 2.5 mL of concentrated sulfuric acid was added directly to the liquid surface in a steady stream. After 15 min, the absorbance was read at 490 nm. The blank was prepared in the same manner, except that 80% ethanol was used as the sample.

### Creating a low oxygen environment during microscopic observations

Whole seedlings were left submerged in water between a slide and coverslip to create an oxygen-limited environment for 45 min and up to 3 h. Observations were taken periodically during this time. Caution was also taken to make sure that no air pockets or air bubbles were trapped around the seedlings, as these hindered the hypoxic effects by prolonging O_2_ availability.

### Fluorescence microscopy

A Leica TCS SP5 confocal microscope was used for visual analysis. Argon (488 nm) and Helium-Neon (543 nm) excitation lasers were used to simultaneously visualize GFP labeled mitochondria (emission band 503–524 nm) and RFP labeled ER (emission band 566–643) while chlorophyll auto-fluorescence was collected between 660 and 750 nm in green tissues as an internal control for cell health. MitotrackerⓇ Orange CMTMRos (Life technologies; cat. No. M-7510) used to stain mitochondria in the *adl2a/drp3a/apm1-1* mutant was excited using 543 nm laser and visualized at 555–622 nm. A 50 W halogen bright field lamp on the epi-fluorescence microscope was used for high light (800 ± 50 μmol m^−2^s^−1^) treatments to induce the fission of elongated and giant mitochondria.

Photo-conversion of mito-mEosFP was carried out as reported earlier (Mathur et al., 2010) using epi-fluorescent lighting through a D filter cube (Leica UV/violet; Ex, BP 355–425; dichroic, 455; Em, LP 470 nm) and a 40 X water-immersion lens (N.A. 0.8). Photo-converting only a subset of mitochondria from green to red permitted assessment of subsequent fusion, which was indicated by the mixing of green and red matrix to form an intermediate orange color.

All confocal images were acquired at a box-size of 1024 × 512 pixels. Three-D (X,Y,Z) stacks maintained a 0.99 μm distance between images whereas the difference between successive images (x,y,t) was maintained at 3.79 s.

### Image analysis and statistics

For mitochondrial length analysis, 10 mitochondria per cell were measured randomly. All experiments consisted of at least four biological and two technical replications. When there was an unequal sample size per treatment, a two-tailed *t*-test was used to determine the significance of results. For equal sample sizes, a Tukey's test of significance was used (Tukey, 1949). Significance was predetermined as having a *p* < 0.05 (95% confidence interval).

The image analysis software, ImageJ (http://imagej.nih.gov/ij/) was used to measure the length of mitochondria and create color profiles using the 3D inspector plugin. All images were cropped and processed for brightness/contrast as complete montages using Adobe Photoshop CS6 (http://www.adobe.com) The layer function in Photoshop was used to introduce text, regions of interest, and color overlays. The 3-dimensional (3D) iso-surface volume rendering of images of mito-GFP RER double transgenics was carried out using IMARIS (v. 6.4.0; Bitplane AG).

## Author contributions

EA and JM co-designed and carried out experiments and wrote the manuscript while KB and NM provided strong support in generating images and plant materials.

## Author note

The author responsible for distribution of materials integral to the findings presented in this article in accordance with the policy described in the Instructions for Authors (www.plantcell.org) is: JM (jmathur@uoguelph.ca).

### Conflict of interest statement

The authors declare that the research was conducted in the absence of any commercial or financial relationships that could be construed as a potential conflict of interest.
