# Supplementary material for: Mitochondrial pleomorphy in plant cells is driven by contiguous ER dynamics
Source: Front Plant Sci. 2015 Sep 24;6:783. doi: 10.3389/fpls.2015.00783 (PMC4585081; doi:10.3389/fpls.2015.00783)

Supplementary Information

Figure 1. Estimation of total soluble sugar [µg/mg of fresh weight (f.w.)] in seedlings grown in the light and dark with or without sucrose. Standard deviations are indicated (4 treatments; 3 technical replicates and n=50 plants per treatment).

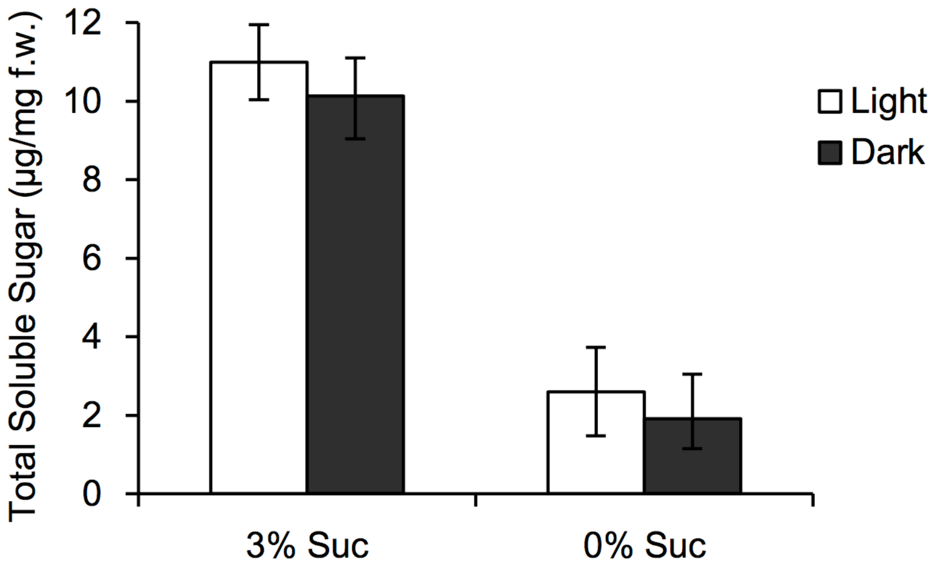

Figure 2. Different mitochondrial forms observed in elongated mitochondria in the cotyledon of fission-impaired *nmt1-2/elm1-1* mutant of Arabidopsis transformed with RFP-ER.

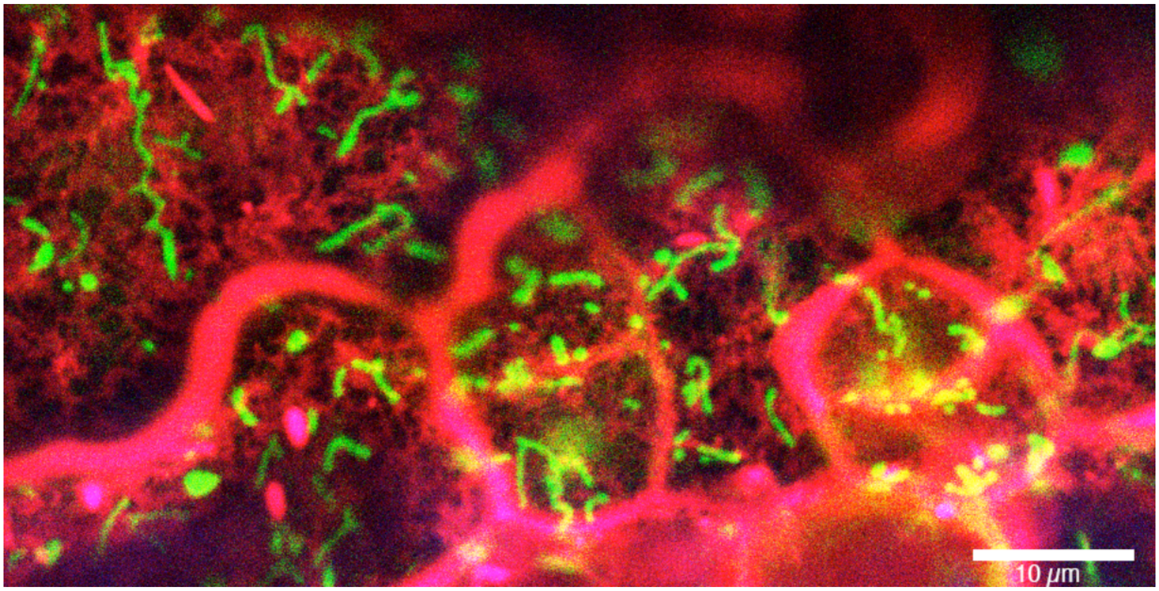

Figure 3. The beads-on-a-string form of a mitochondrion observed in a hypocotyl cell of the *adl2a/apm1-1* mutant sedling stained with Mitotracker®Orange CMTMRos.

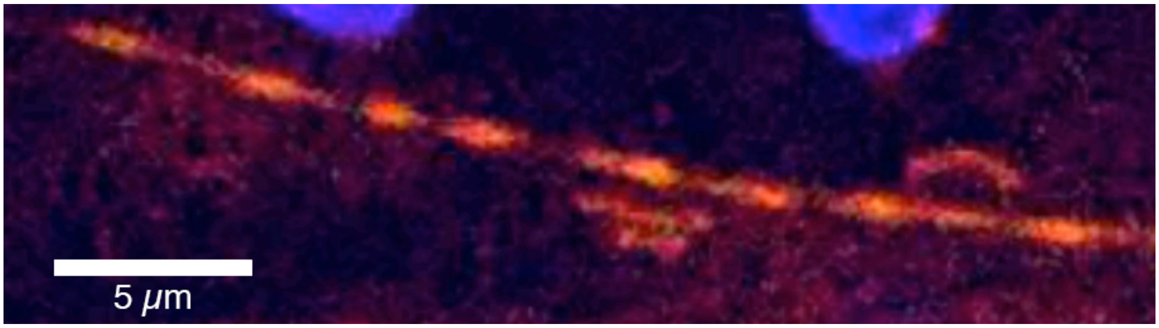

Supplement: Supplementary file 7 [file Image1.PDF]
