# Supplementary material for: Mitochondrial pleomorphy in plant cells is driven by contiguous ER dynamics
Source: Front Plant Sci. 2015 Sep 24;6:783. doi: 10.3389/fpls.2015.00783 (PMC4585081; doi:10.3389/fpls.2015.00783)
Supplement: Supplementary file 8 [file Image2.PDF]

Supplemental QuickTime Movie Legends:

All time lapse images were taken 4 seconds apart at a box size of 1024 X 512 pixels before being cropped, threaded using Quick time, and are played at 6 frames per second.

Movie 1. Close interactions over between an elongated photo-converted (red) and a non-photo-converted mitochondrion (green) over several minutes resulted in fusion and the formation of a giant mitochondrion with an intermediate, yellow-orange colour

Movie 2. A time-lapse sequence from a seedling expressing mito-GFP and RER spanning about 1.5 minutes shows the fission of an elongated mitochondrion into three smaller units. Note the alignment of the two organelles and the displacement of the neighbouring ER (red). Several contorted mitochondria are observed in the left side of the movie and will undergo fission subsequently.

Movie 3. A time-lapse sequence that captured 2.8 minutes of mitochondrial (in green) and ER (in red) activity in a cell exposed to light after being kept under hypoxia for nearly 45 minutes shows the stretching of a mitochondrion followed by its fission into three portions. Fission occurs at the thin neck (suggestive of a matrixule) and results in the three portions snapping back to a nearly circular form confined to the expanded ER cisternae harboring them. A single yellow fluorescent peroxisome is also seen in the sequence but does not exhibit an extended shape like the mitochondrion.

Movie 4. A time-lapse image sequence covering 8.5 minutes shows the beads-on-a-string and other mitochondrial forms produced as the dynamic ER pulls on expanded mitochondria.

Movie 5. Time-lapse sequence spanning a total of 17.5 minutes from a light grown seedling of the *nmt1-2/elm1-1* expressing RER shows the aggregation and fusion of punctate mitochondria to form the elongated mitochondria characteristic of this fission-impaired mutant.

Movie 6. Snapshots from a time-lapse series covering nearly 16.5 minutes of activity resumed in a cell. The cell had developed expanded mitochondria and ER cisternae after nearly 45 minutes of hypoxia and resumption of dynamic behaviour for both organelles occurred following a five-minute exposure to light. Mitochondrial blobs started extending and stretching and eventually broke up as contiguous ER polygons reorganized. A matrixule–like projection extended from an expanded mitochondrion on the left side of the movie preceded a fission and snap-back event.
